# Matrix-M™ adjuvant enhances immunogenicity of both protein- and modified vaccinia virus Ankara-based influenza vaccines in mice

**DOI:** 10.1007/s12026-018-8991-x

**Published:** 2018-03-28

**Authors:** Sofia E. Magnusson, Arwen F. Altenburg, Karin Lövgren Bengtsson, Fons Bosman, Rory D. de Vries, Guus F. Rimmelzwaan, Linda Stertman

**Affiliations:** 1 Novavax AB, Kungsgatan 109, SE-75318 Uppsala, Sweden; 2 Department of Viroscience, Erasmus MC, Rotterdam, The Netherlands; 3 Amatsigroup NV, Biologicals Unit, Ghent, Belgium; 4 Present Address: Research Center for Emerging Infections and Zoonoses, University of Veterinary Medicine, Hannover, Germany

**Keywords:** Matrix-M™ adjuvant, Influenza virus, MVA, Vaccine, Immunogenicity

## Abstract

Influenza viruses continuously circulate in the human population and escape recognition by virus neutralizing antibodies induced by prior infection or vaccination through accumulation of mutations in the surface proteins hemagglutinin (HA) and neuraminidase (NA). Various strategies to develop a vaccine that provides broad protection against different influenza A viruses are under investigation, including use of recombinant (*r*) viral vectors and adjuvants. The replication-deficient modified vaccinia virus Ankara (MVA) is a promising vaccine vector that efficiently induces B and T cell responses specific for the antigen of interest. It is assumed that live vaccine vectors do not require an adjuvant to be immunogenic as the vector already mediates recruitment and activation of immune cells. To address this topic, BALB/c mice were vaccinated with either protein- or rMVA-based HA influenza vaccines, formulated with or without the saponin-based Matrix-M™ adjuvant. Co-formulation with Matrix-M significantly increased HA vaccine immunogenicity, resulting in antigen-specific humoral and cellular immune responses comparable to those induced by unadjuvanted rMVA-HA. Of special interest, rMVA-HA immunogenicity was also enhanced by addition of Matrix-M, demonstrated by enhanced HA inhibition antibody titres and cellular immune responses. Matrix-M added to either protein- or rMVA-based HA vaccines mediated recruitment and activation of antigen-presenting cells and lymphocytes to the draining lymph node 24 and 48 h post-vaccination. Taken together, these results suggest that adjuvants can be used not only with protein-based vaccines but also in combination with rMVA to increase vaccine immunogenicity, which may be a step forward to generate new and more effective influenza vaccines.

**Electronic supplementary material:**

The online version of this article (10.1007/s12026-018-8991-x) contains supplementary material, which is available to authorized users.

## Introduction

Influenza A (H1N1 and H3N2) and B viruses cause respiratory tract infections and are responsible for substantial morbidity and mortality during seasonal epidemics, particularly in patients at high risk, such as the elderly. Due to accumulation of mutations in the surface proteins hemagglutinin (HA) and neuraminidase (NA), the antigenic properties of the virus change continuously, resulting in escape from recognition by neutralizing antibodies induced by prior infection or vaccination [1–3]. Furthermore, avian influenza viruses of various subtypes have been shown to infect humans sporadically [4–6]. Since virus neutralizing antibodies to these viruses are virtually absent in the human population, they are considered to have pandemic potential.

Currently used inactivated influenza vaccines contain components from seasonal influenza viruses and aim at the induction of HA-specific neutralizing antibodies [7, 8]. Despite annual assessment of virus strains to be included in the seasonal influenza vaccine, a mismatch between circulating influenza viruses and the vaccine strains occasionally occurs, resulting in reduced vaccine effectiveness [9–11]. Furthermore, novel tailor-made influenza vaccines need to be developed momentarily in case of an influenza virus pandemic. Clearly, there is a need for improved influenza vaccines that can be produced rapidly and are highly immunogenic, inducing broadly protective immunity to various influenza viruses.

Presently, novel vaccine targets, adjuvants, and delivery systems are under investigation to develop “next-generation” influenza vaccines. Recombinant viral vaccine vectors, including modified vaccinia virus Ankara (MVA) and adenoviruses, can be used to drive expression of any antigen of interest, resulting in efficient induction of antigen-specific B and T lymphocyte responses [12, 13]. Particularly, MVA is considered to be of interest since it has an excellent safety record in humans, including immunocompromised individuals [12–15]. Design and rescue of recombinant (r)MVA expressing one or more antigens are relatively easy and can be performed rapidly, and large numbers of vaccine doses can be produced [12]. Previously, several rMVA vaccines expressing HA from various influenza viruses have been evaluated in vitro and in vivo and have shown to be immunogenic and capable of inducing protective immunity against homologous and heterologous influenza virus infections [13].

Another approach to enhance influenza vaccine immunogenicity is the use of adjuvants [16]. Adjuvants such as MF59, AS03, Alum, ISCOMATRIX®, and Matrix-M™ adjuvant have successfully been evaluated in clinical trials in combination with seasonal and pandemic influenza vaccines, including inactivated whole virus, split-virion, virosomal, and virus-like particle vaccines [17–23]. Furthermore, MF59 and AS03 have been approved for use in a seasonal and pre-pandemic A(H5N1) influenza vaccine, respectively [24]. Matrix-M adjuvant, made of *Quillaja saponins* formulated with cholesterol and phospholipids into nanoparticles, is known to augment Th1 and Th2 responses, induce antibodies of multiple subclasses, enhance immune cell trafficking, and allow antigen dose-sparing [25–31]. Importantly, Matrix-M-adjuvanted vaccines have been shown to have an acceptable safety profile in clinical trials [21–23]. Compared to other adjuvants, Matrix-M performed as well or better in combination with influenza vaccines in mice [27, 32].

In contrast to protein-based vaccines, which are poorly immunogenic without adjuvant, vector-based vaccines are generally thought not to require adjuvants due to the intrinsic adjuvant activity of the vector backbone [33]. However, recently, it was shown that immunogenicity of malaria and Rift Valley Fever virus antigens expressed from adenovirus or MVA was improved by addition of Matrix-M [34, 35]. In the present study, we show that the immunogenicity of both HA protein- and MVA-based influenza vaccines was enhanced by Matrix-M adjuvant. Co-formulation of either vaccine with Matrix-M adjuvant increased absolute immune cell numbers and activation in the lymph node (LN) draining the site of vaccination up to 48 h after injection.

## Material and methods

### Matrix-M™ adjuvant

Novavax’s proprietary Matrix-M™ adjuvant consists of two individually formed 40-nm-sized particles, each with a different and well-characterized saponin fraction (Fraction-A and Fraction-C). The Matrix-A and -C particles are formed by formulating purified saponin from the tree *Quillaja saponaria* Molina with cholesterol and phospholipid [36].

### Preparation of HA protein

Recombinant HA (H1N1, A/Puerto Rico/8/34 [PR8]) was produced in HEK293F cells as an amino-terminal His-tagged fusion protein containing a linker sequence (PGGPGS) and mcaspase3 cleavage site (DELD) but lacking the HA transmembrane sequence. The secreted (His6-PGGPGSDELD)-HA protein was purified by metal affinity chromatography. After mcaspase treatment (E/S mass ratio 1/30), the protein solution was loaded on a Superdex G200 gel filtration column and the HA were fractions pooled. Analysis by SDS-PAGE/CBB staining and western blot showed that mature (cleaved) HA protein was obtained with a purity of at least 90%.

### Generation of rMVA-HA

rMVA expressing HA under control of the early/late vaccinia virus promotor PsynII using the MVA clonal isolate F6 was produced as previously described [37]. In short, the codon-optimized HA nucleotide sequence (PR8, accession number CY033577) was purchased from Baseclear B.V. and rMVA was prepared through mCherry-dependent plaque selection in chicken embryo fibroblasts (CEF). To generate a final vaccine preparation, the virus was amplified in CEF, purified by ultracentrifugation through 36% sucrose, and reconstituted in 120-mM NaCl and 10-mM Tris-HCl pH 7.4. rMVA-HA constructs were characterized by PCR, sequencing, plaque titration, western blot, and in vitro infection of various cell types.

### Vaccination of BALB/c mice

Specified pathogen-free female BALB/c mice (8–10 weeks old) were purchased from Charles River Laboratories (Germany). Animals were housed in Makrolon type 3 cages, had access to food and water ad libitum, and animal welfare was observed daily. All experiments were conducted in compliance with European guidelines and the protocol approved by an independent animal experimentation ethical review committee (Uppsala djurförsöksetiska nämnd). Two separate experiments were performed. In the first experiment, mice (*n* = 5 or 8/group) received two vaccinations with 10^8^ plaque forming units (PFU) of rMVA-HA or 1 or 10 μg of HA, formulated with or without 5-μg Matrix-M, at a 4-week interval. All vaccines were administered subcutaneously (s.c.) in 100 μL at the base of the tail. Blood samples were obtained at day 21 and day 42. Spleens were collected in PBS during necropsy. In the second experiment, mice (*n* = 30/group) were immunized intramuscularly (i.m.) in the hind leg with a volume of 50 μL containing 10^8^-PFU rMVA-HA or 10-μg HA, with or without 5-μg Matrix-M. The inguinal LN draining the hind leg muscle was collected in PBS at 4, 24, or 48 h post-vaccination (*n* = 10/group/timepoint).

### Detection of IgG1 and IgG2a HA-specific serum antibodies

Quantification of HA-specific IgG1 and IgG2a antibodies was performed by ELISA as described previously [27]. Briefly, 96-well Maxisorp microplates (Nunc) coated overnight (O/N) at 4 °C with 50-ng/well HA protein in 0.05-M carbonate/bicarbonate buffer (Sigma-Aldrich). Serum from untreated mice and HA-positive mouse serum was used as negative or positive control, respectively. IgG1 and IgG2a anti-HA titers were calculated using a four-parameter logistic equation (Softmax software, Molecular Devices). The inflection point of the titration curve (EC_50_ value) was taken as titer value.

### Hemagglutination inhibition (HI) assay

Sera were treated with a receptor-destroying enzyme (filtrate of *Vibrio cholerae*) O/N at 37 °C followed by heat inactivation for 1 h at 56 °C. Sera were titrated in a twofold serial dilution. The HI assay was performed in duplicate following a standard protocol with 1% turkey erythrocytes and four HA-units of influenza virus PR8, as described previously [38].

### Fluorospot analysis of antigen-stimulated splenocytes

Single-cell suspensions from spleens of individual mice, prepared as previously described [27], were seeded on filter plates coated with anti-interleukin 2 (IL-2) and -interferon gamma (IFN-γ) capture antibodies (Mabtech), at 0.25 × 10^6^ cells/well in culture medium (Roswell Park Memorial Institute, Sigma-Aldrich) supplemented with 10% heat-inactivated fetal bovine serum (Sigma-Aldrich) and 100-U/ml penicillin, 100-μg/ml streptomycin, and 2-mM L-glutamin (Sigma-Aldrich), followed by stimulation with 0.5-μg/well HA protein. Concanavalin A (Sigma-Aldrich) and culture medium were used as positive and negative controls, respectively. Triplicate samples were incubated for 18 h at 37 °C and IL-2 and/or IFN-γ spots were developed according to the manufacturer’s instructions (Mabtech). Spots were detected using an AID ELR02 ELISpot reader (Autoimmune Diagnostika GmbH).

### Flow cytometry analysis of immune cells in the dLN

Single-cell suspensions from the draining (d)LN, prepared as described previously [27], were stained with FVS780 (BD Biosciences) for 15 min at room temperature to exclude dead cells during analysis. Cells were washed and resuspended in FACS buffer (PBS with 0.5% bovine serum albumin, 2-mM EDTA, and 0.1% NaN_3_,) and incubated for 20 min at 4 °C with anti-mouse CD16/CD32 (2.4G2, BD Biosciences). 5 × 10^5^ cells/well were transferred to a 96-well microtiter plate (Nunc) and incubated with anti-mouse CD86:FITC (GL1), I-A/I-E:BV605 (M5/114), CD8a:BV650 (53-6.7), CD19:PerCP-Cy5.5 (1D3), CD3e:PerCP-Cy5.5 (145-2C11), Ly-6G:BV786 (1A8) (all BD Biosciences), CD169:AlexaFluor647 (3D6.112), CD11c:BV650 (N418), Ly-6C:APC (HK1.4), CD69:BV421 (H1.2F3), CD3e:PE (145-2C11), F4/80:BV421 (BM8), CD11b:PE (M1/70), CD49b:APC (DX5), and CD4:BV785 (RM4-5) (all Nordic Biosite) for 30 min at 4 °C. Fluorescence minus one controls were prepared for each antibody in all antibody panels at acquisition timepoints. Samples were analyzed on FACSCelesta with FACSDiva software (BD Biosciences).

### Statistical analysis

Serological and cellular data were analyzed using one-way ANOVA with Tukey’s post-test for multiple comparisons or Kruskal-Wallis with Dunn’s multiple comparisons test when applicable.

## Results

### Addition of Matrix-M adjuvant enhanced HA-specific humoral responses

**Fig. 1:**
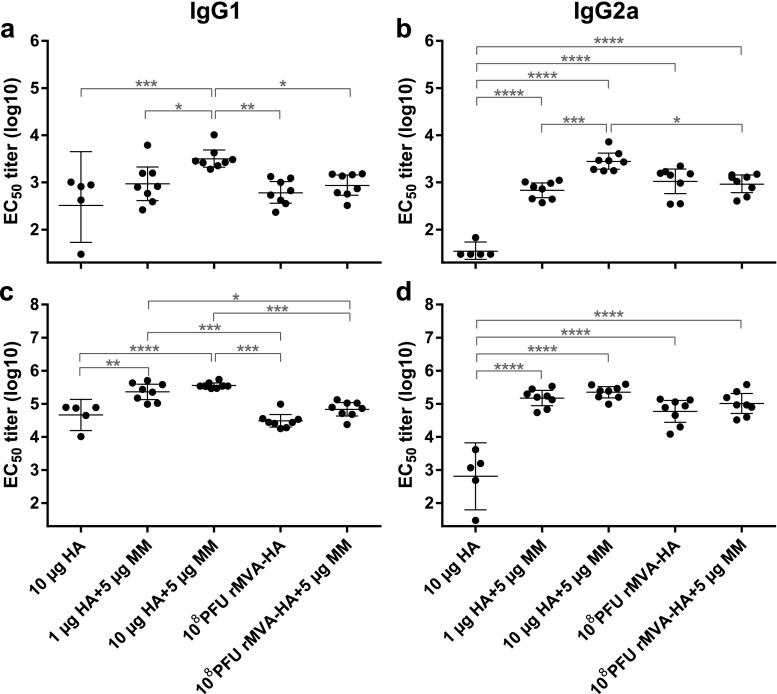
HA-specific antibody responses induced after vaccination with HA protein or rMVA-HA with or without Matrix-M adjuvant IgG1 (**a**) and IgG2a (**b**) HA-specific antibody responses 21 days after the primary vaccination. **c**–**d** IgG1 and IgG2a HA-specific antibody responses 14 days after the booster vaccination. IgG1 (**a**, **c**) or IgG2a (**b**, **d**) serum antibodies were detected by ELISA using purified HA protein and anti-IgG1 or anti-IgG2a HRP-conjugated antibodies. Data is shown as mean ± 95% confidence interval (CI). *p < 0.05; **p < 0.01; ***p < 0.001; ****p < 0.0001. MM = Matrix-M adjuvant

To assess HA-specific antibody responses after vaccination with either protein- or rMVA-based vaccines with or without Matrix-M adjuvant, mice were immunized at days 0 and 28. At 21 days after the primary vaccination, HA-specific serum antibody responses were detected in all groups. Strongest antibody responses of both IgG1 and IgG2a were detected after vaccination with 10-μg HA adjuvanted with Matrix-M. Without adjuvant, protein-based HA vaccines induced IgG1 and IgG2a responses inefficiently. After one immunization with rMVA-HA, HA-specific IgG1 and IgG2a antibody responses were induced, which were not further enhanced by Matrix-M addition (Fig. 1a–b). Fourteen days after the second vaccination, HA-specific IgG1 and IgG2a levels were boosted in all groups (Fig. 1c–d). Addition of Matrix-M to both HA doses significantly increased IgG1 responses compared to unadjuvanted HA or rMVA-HA vaccination with or without adjuvant (Fig. 1c). The IgG2a responses after the second vaccination were comparable between adjuvanted HA groups and both rMVA-HA vaccine groups and were elevated compared to the unadjuvanted HA group (Fig. 1d). Use of Matrix-M did not increase IgG1 or IgG2a responses after the second vaccination with rMVA-HA.

**Fig. 2:**
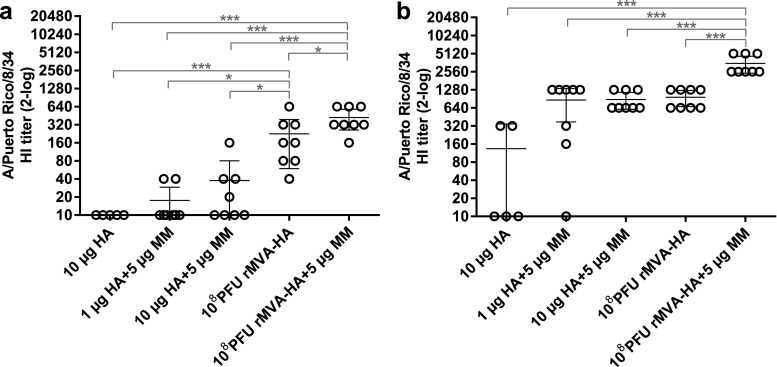
Induction of HA-specific HI antibody responses after vaccination with rMVA-HA adjuvanted with Matrix-M HI serum antibody responses against influenza virus A/Puerto Rico/8/34 (H1N1) was measured 21 days after the primary (**a**) or 14 days after the booster (**b**) vaccination. Data is shown as mean ± 95% CI. *p < 0.05; **p < 0.01; ***p < 0.001. MM = Matrix-M adjuvant

Next, HI antibody titers were determined, which is considered a good proxy for the virus-neutralizing antibody response. In contrast to the IgG1 and IgG2a responses detected after primary vaccination, mice vaccinated with rMVA-HA displayed significantly elevated HI titers compared to those vaccinated with HA, regardless of antigen dose and use of adjuvant (Fig. 2a). After booster vaccination, HI titers were detected in mice receiving Matrix-M-adjuvanted HA, whereas lower HI titers were detected in only two out of five mice receiving unadjuvanted HA. After two immunizations, adjuvanted HA induced similar HI titers as rMVA-HA vaccination (Fig. 2b). Of special interest, higher HI titers were observed in mice vaccinated with adjuvanted rMVA-HA compared to mice that received unadjuvanted rMVA-HA.

### Addition of Matrix-M adjuvant enhanced HA-specific cellular responses

**Fig. 3:**
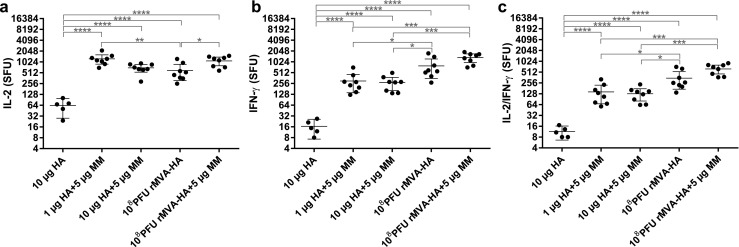
Enhanced HA-specific splenocyte responses by Matrix-M-adjuvanted vaccine spleens obtained 14 days after the booster vaccination were stimulated with purified HA protein and the number of IL-2 (**a**), IFN-γ (**b**), and IL-2/IFN-γ (**c**) producing splenocytes was determined in spot forming units (SFU)/10^6^ cells by Fluorospot assay. Samples were tested in triplicate. The mean ± 95% CI of each group is indicated. *p < 0.05; **p < 0.01; ***p < 0.001; ****p < 0.0001. MM = Matrix-M adjuvant

To investigate HA-specific T lymphocyte responses after booster vaccination, splenocytes were stimulated with HA protein and the number of IL-2 and/or IFN-γ producing cells was measured. Matrix-M-adjuvanted HA and rMVA-HA, with or without adjuvant, induced significantly more IL-2 and/or IFN-γ producing splenocytes than HA alone. Unadjuvanted HA hardly induced any IL-2 and/or IFN-γ splenocyte responses (Fig. 3a–c). In contrast, co-formulation of HA with Matrix-M resulted in higher IL-2 responses compared to rMVA-HA, adjuvanted or not, whereas rMVA-HA induced higher IFN-γ responses compared to adjuvanted HA (Fig. 3a–c). Mice vaccinated with Matrix-M-adjuvanted rMVA-HA displayed stronger IL-2, IFN-γ, and IL-2/IFN-γ double-positive responses compared those receiving unadjuvanted rMVA-HA, although the differences were exclusively statistically significant for the IL-2 response (Fig. 3a–c).

### Matrix-M-adjuvanted vaccines increased cell numbers in the dLN

**Fig. 4:**
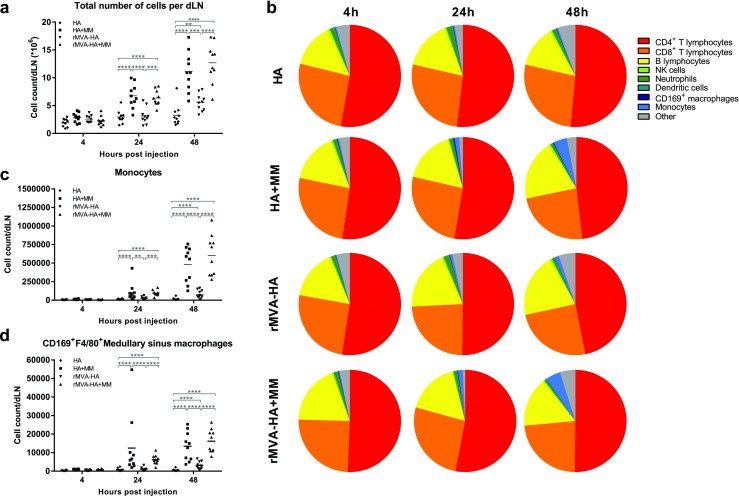
Matrix-M-adjuvanted influenza vaccines induce influx of immune cells in the dLN with maintained composition of cellular subsets except for an increased monocyte population. **a** The total number of cells per dLN. **b** Contribution (%) of the indicated cellular subsets in the dLN was measured by flow cytometry at 4, 24, or 48 h after i.m. vaccination. **c**–**d** Total cell count of CD11b^+^Ly6C^+^ monocytes (**c**) and CD169^+^F4/80^+^ medullary sinus macrophages (**d**) were determined by flow cytometry. Data are shown as mean of 10 mice per group. *p < 0.05; **p < 0.01; ***p < 0.001; ****p < 0.0001. MM = Matrix-M adjuvant

It is known that injection of Matrix-M adjuvant alone leads to influx of several types of immune cells to the dLN at early timepoints, and this effect has been associated with increased antigen-specific immune responses [27, 28]. As addition of Matrix-M to either protein- or MVA-based vaccines increased both HA-specific humoral and cellular immune responses, we wanted to evaluate the early cellular immune response in the dLN to explore possible differences when combining the adjuvant with the respective vaccine type. Mice were vaccinated i.m. with HA or rMVA-HA, with or without Matrix-M, and dLNs were collected 4, 24, and 48 h post-vaccination. At 4 h post-vaccination, the mean number of total cells in the dLN of all vaccine groups was similar (Fig. 4a). In contrast, after 24 and 48 h, the total cell count per dLN of mice vaccinated with adjuvanted HA or rMVA-HA showed more than a twofold increase compared to the unadjuvanted groups (Fig. 4a). Of note, although not as strong as the adjuvanted vaccines, vaccination with unadjuvanted rMVA-HA also resulted an increase in cell count per dLN compared to HA alone.

The relative contribution of different cell populations (Supplementary Fig. 1) to the total cell number in each dLN (*n* = 10/group) was determined. At all timepoints, regardless of vaccine type or use of adjuvant, CD4^+^ T lymphocytes comprised the largest proportion of the total cell population, followed by CD8^+^ T and B lymphocytes (Fig. 4b). No significant difference in the percentage of neutrophils, macrophages, NK cells, or DCs was observed (Fig. 4b). In contrast, mice vaccinated with Matrix-M-adjuvanted HA or rMVA-HA and to a lesser extent, unadjuvanted rMVA-HA showed a strong increase in proportion and total number of monocytes in the dLN over time, indicating recruitment and/or proliferation compared to HA alone (Fig. 4b–c). Notably, the number of medullary sinus macrophages (CD169^+^F4/80^+^) was increased by Matrix-M-adjuvanted HA and rMVA-HA at 24 and 48 h after vaccination compared to unadjuvanted vaccine preparations (Fig. 4d). At 48 h, the unadjuvanted rMVA-HA group also showed an increase in medullary sinus macrophages compared to the unadjuvanted HA group, but to a lesser extent than the adjuvanted vaccines.

### Cell activation in the dLN after vaccination with Matrix-M-adjuvanted vaccine preparations

**Fig. 5:**
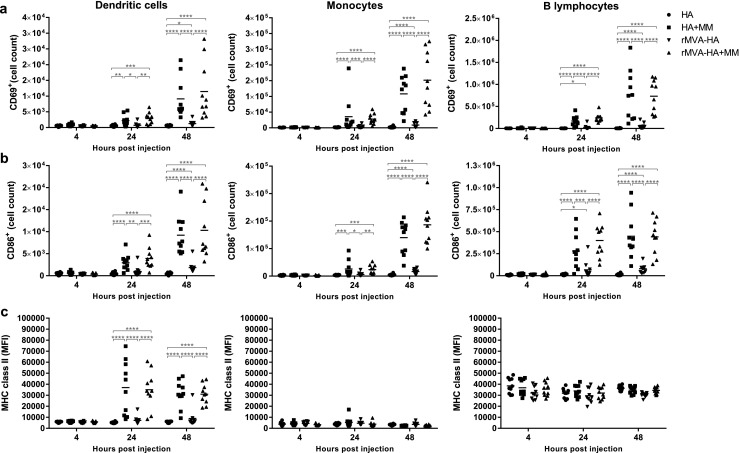
Increased activation of APCs in the dLN 24 and 48 h after vaccination with Matrix-M-adjuvanted influenza HA vaccines. **a**–**b** The number of CD69^+^ or CD86^+^ DCs, monocytes, and B lymphocytes recruited to the dLN was measured by flow cytometry 4, 24, and 48 h after i.m. injection of the respective vaccine. **c** The mean fluorescence intensity (MFI) of MHC class II of on DCs, monocytes, and B lymphocytes in the dLN was measured by flow cytometry 4, 24, and 48 h post-injection. Data are shown as mean of 10 mice per group. *p < 0.05; **p < 0.01; ***p < 0.001; ****p < 0.0001. MM = Matrix-M adjuvant

**Fig. 6:**
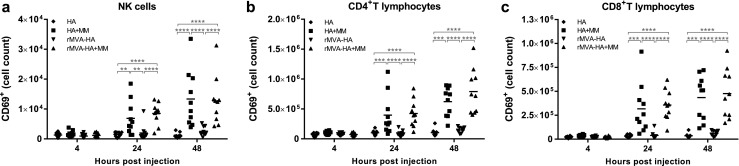
Increased number of activated NK- and T lymphocytes in the dLN at 24 and 48 h post-vaccination with Matrix-M-adjuvanted influenza HA vaccines The number of CD69^+^ NK cells (**a**), CD4^+^ (**b**), and CD8^+^ (**c**) T lymphocytes in the dLN was measured by flow cytometry 4, 24, and 48 h after i.m. injection of the respective vaccines. Data are shown as mean of 10 mice per group. *p < 0.05; **p < 0.01; ***p < 0.001; ****p < 0.0001. MM = Matrix-M adjuvant

Activation of different cellular subsets was investigated by measuring expression of CD69 (early activation marker [39]), CD86 (T lymphocyte co-stimulatory signal [40]), and/or MHC class II (often upregulated on antigen-presenting cells (APC) after activation). Expression of both CD69 and CD86 was upregulated on DCs, monocytes, and B lymphocytes 24 and 48 h after vaccination with either Matrix-M-adjuvanted HA or rMVA-HA compared to the respective unadjuvanted vaccine preparation (Fig. 5a–b). Unadjuvanted rMVA-HA also induced an increase in CD69^+^ and CD86^+^ DCs, monocytes, and B lymphocytes compared to unadjuvanted HA at 24 and 48 h post-vaccination, however, only to a limited extent compared to the adjuvanted vaccines (Fig. 5a–b). In addition to the increase in CD69^+^ and CD86^+^ APCs, MHC class II expression in DCs was elevated at 24 and 48 h post-vaccination with the adjuvanted vaccine preparations (Fig. 5c). CD69 expression was also assessed for T lymphocytes and NK cells. The number of CD69^+^ NK cells and T lymphocytes was significantly increased after vaccination with Matrix-M-adjuvanted HA, or rMVA-HA, compared to their unadjuvanted counterparts (Fig. 6a–c).

Altogether, rMVA-HA induced relatively more activation, recruitment, and/or proliferation of APC and lymphocytes compared to unadjuvanted HA. However, addition of Matrix-M adjuvant to either protein- or MVA-based HA vaccines significantly increased activation and recruitment and/or proliferation for both vaccine preparations.

## Discussion

Adjuvants increase vaccine immunogenicity via different mechanisms, including antigen delivery and general activation of innate immune responses [41]. Although use of adjuvants for protein-based vaccines is well established and essential for efficient immune responses, addition of adjuvants to vector-based influenza vaccines has not been previously studied. Here, the immunogenicity of influenza virus HA and rMVA-HA vaccines was tested in the presence and absence of Matrix-M adjuvant. Even if unadjuvanted rMVA-HA was more immunogenic than unadjuvanted HA, co-formulation of either vaccine preparation with Matrix-M enhanced HA-specific immune responses and increased the cell number and activation in the dLN.

For induction of proper HA-specific antibody responses of IgG1 (indicative of Th2 responses) or IgG2a (indicative of Th1 responses) subclasses, addition of Matrix-M adjuvant to HA was required, but not to rMVA-HA. After two immunizations, adjuvanted HA induced significantly higher IgG1 antibody responses than rMVA-HA, whereas IgG2a antibody responses were similar. This is in line with previously published data showing that MVA-based vaccines preferentially induce Th1 responses [12, 13]. The observed potentiating effect of Matrix-M on the IgG2a antibody responses has been shown previously with various vaccine preparations in mice [27, 29, 31]. Induction of potent IgG2a responses bares relevance, as murine IgG2 has key immunological effector functions, such as enhanced FcγR binding important for protection against viral infection [42]. Accordingly, passive immunization with HA stalk-specific IgG2a antibodies has shown to protect mice against influenza virus infection, while HA stalk-specific IgG1 antibodies did not [43]. To induce functional antibodies, a single vaccination with rMVA-HA was sufficient for generating acceptable HI antibody titers, whereas for HA, regardless of adjuvantation, two vaccinations were required. This may reflect a better conformational integrity of HA expressed in vivo by rMVA-HA. Strikingly, addition of Matrix-M adjuvant to the rMVA-HA vaccine significantly increased the HI antibody response after both prime and booster vaccination, in spite of the adjuvant having no clear effect on the HA-specific IgG1 and IgG2a titers for the rMVA-HA vaccine.

Addition of Matrix-M adjuvant to HA potentiated HA-specific IFN-γ and IL-2/IFN-γ cellular responses significantly compared to HA alone, in concordance with previous studies [29–31, 44]. Interestingly, mice vaccinated with rMVA-HA showed stronger IFN-γ responses than those vaccinated with adjuvanted HA. The rMVA-HA-induced cellular responses could be even further increased by addition of Matrix-M. Although the phenotype of the responding cells was not determined, these are most likely CD4^+^ T lymphocytes as exogenous HA protein was used for stimulation.

It was previously shown in mice that injection with Matrix-M adjuvant alone led to increased numbers of activated immune cells in the dLN compared to PBS or other adjuvants [27, 28]. Here, the absolute number of cells in the dLN of mice vaccinated with adjuvanted HA or rMVA-HA vaccines was significantly higher compared to mice vaccinated with unadjuvanted vaccines 24 and 48 h post-vaccination, indicative of proliferation and/or recruitment. The dLN cell composition was stable, except for an increase in monocytes after vaccination with adjuvanted vaccine preparations. Recruited monocytes could mature into DCs and/or macrophages in situ and subsequently act as professional APC [45], potentially improving vaccine efficacy. This could also be the effect of the increase in CD169^+^ medullary sinus macrophages, also detected in the dLN after injection with Matrix-M-adjuvanted vaccines. Recently, CD169^+^ macrophages were shown to be important for the adjuvant properties of the saponin-based adjuvant QS21 [46]. CD169^+^ macrophages have been shown to transport antigens trapped inside the LN follicle to B lymphocytes and can cross-present antigen directly to CD8^+^ T lymphocytes [47–49]. Thus, the increase in CD169^+^ macrophages may play a role in the improved adaptive immune responses induced by Matrix-M-adjuvanted vaccines.

Vaccination with unadjuvanted rMVA-HA induced a relative increase in monocytes accompanied by increased activation of CD86^+^ DC, CD86^+^ B lymphocytes, and CD169^+^ macrophages, confirming that MVA has intrinsic adjuvant properties. Of interest, it was recently shown that APCs can be infected by MVA and detected in the dLN of various species including non-human primates [50]. Thus, the observed adjuvant capacities of MVA may be explained by direct infection of APCs, which travel to the dLN, shaping the immune response.

In conclusion, our results show that influenza vaccines based on recombinant HA protein or rMVA-HA can be potentiated by Matrix-M adjuvant, resulting in improved humoral and cellular responses. This is potentially mediated by recruitment and activation of immune cells in the dLN. Combination of a vector-based vaccine with Matrix-M adjuvant might prove a promising step towards next-generation influenza vaccines.

## Electronic supplementary material

ESM 1 (DOCX 354 kb)
