# Supplementary material for: Matrix-M™ adjuvant enhances immunogenicity of both protein- and modified vaccinia virus Ankara-based influenza vaccines in mice
Source: Immunol Res. 2018 Mar 28;66(2):224–33. doi: 10.1007/s12026-018-8991-x (PMC5899102; doi:10.1007/s12026-018-8991-x)
Supplement: Supplementary file 1 — (DOCX 354 kb) [file 12026_2018_8991_MOESM1_ESM.docx]

**Supplementary Figure 1**

**
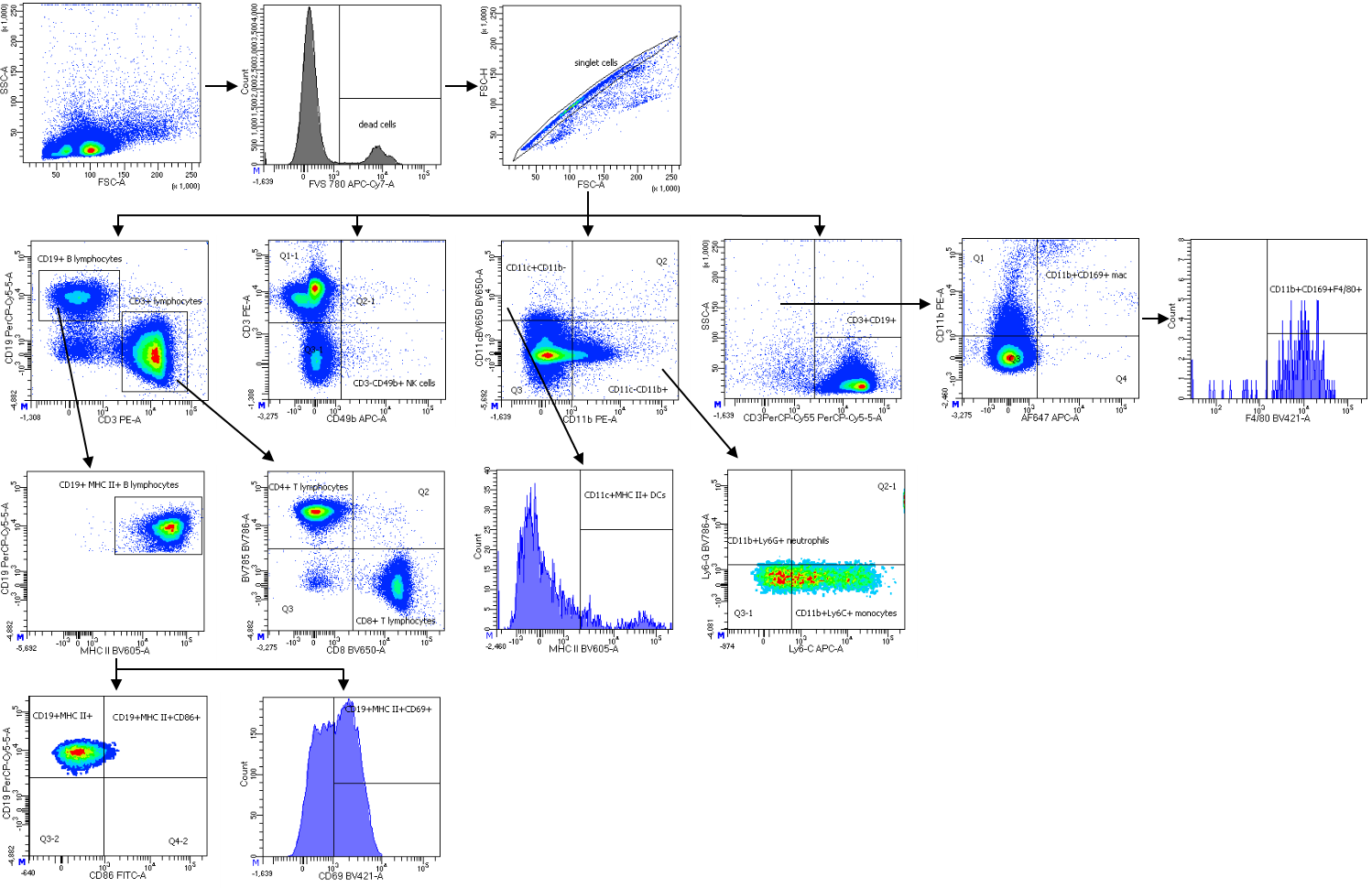
**

**Supplementary Figure 1. Gating strategy to define different immune cell populations in the draining lymph node** Live cells were defined based on being gated as negative for FVS 780. Singlet cells were then selected from the live cells. Subsequently, from the singlet cells CD3^+^ and CD19^+^ cells were selected and B lymphocytes were further defined as double positive for CD19 and MHC class II expression. The B lymphocytes were further characterized for CD86 expression and/or CD69 expression. All defined cell populations were gated in the same way for CD86- and/or CD69 expression. The CD3^+^ cells were further defined as CD4^+^ lymphocytes or CD8^+^ lymphocytes. From the singlet gate NK cells were defined as CD3^-^CD49b^+^. From the singlet gate, cells were selected for CD11c- or CD11b expression and the CD11c^+^ cells further selected for MHC class II expression and defined as dendritic cells. The CD11b^+^ cells were further defined into either Ly6G^+^ cells, i.e. neutrophils or Ly6C^+^ cells, i.e. monocytes. Furthermore from the singlet cells, cells were selected as non-CD3^+^CD19^+^ cells and these cells were further selected for CD11b- and CD169 expression and defined as subcapsular sinus macrophages and further defined as medullary sinus macrophages based on F4/80 expression. Data is shown from a mouse vaccinated with Matrix-M adjuvanted HA protein that is representative of the other mice and groups from the experiment.
